# Total Duplication of the Femur: Case Report with 23 Years Follow-up

**DOI:** 10.1055/s-0041-1735139

**Published:** 2021-10-01

**Authors:** Rafael Garcia de Oliveira, Giampaulo Marcelo Catelan, Cicero Ricardo Gomes

**Affiliations:** 1 Departamento de Ortopedia, Rede Sarah de Hospitais de Reabilitação, Brasília, DF, Brasil

**Keywords:** dysmetria, femur, limb deformities, congenital, scoliosis

## Abstract

Femur duplication is very rare, and the few cases described are of partial, proximal or distal duplications. Based on our literature review, the present is the first report of a case of total duplication. The patient was followed up, without surgical treatment, for 23 years. At the last visit, the patient complained of low-back pain and marked discrepancy in the lower limbs. A clinic-radiographic evaluation showed a right knee with two separate femoral condyles that articulated with the proximal tibia, a hyporadioactive patella, and a limitation in knee flexion at 95°. The patient presented lower-limb dysmetria, measuring 17 cm, resulting in pelvic obliquity and dextroconvex scoliosis. The patient received a 16 cm orthoprosthesis that improved the gait pattern and resolved the complaint of low-back pain.

## Introduction

Congenital short femur is the most common femoral deformity. Some authors have described rare cases of distal femoral bifurcation, which is characteristic of the Gollop-Wolfgang complex, a rare genetic syndrome usually associated with deformities of the ipsilateral limb, such as tibial aplasia, hypoplasia, or congenital talipes equinovarus, most commonly known as clubfoot. 1 2 3 4 There was also a case of proximal femoral duplication reported in 2009; however, total femoral duplications have never been described in the literature.

## Case Report

**Fig. 1:**
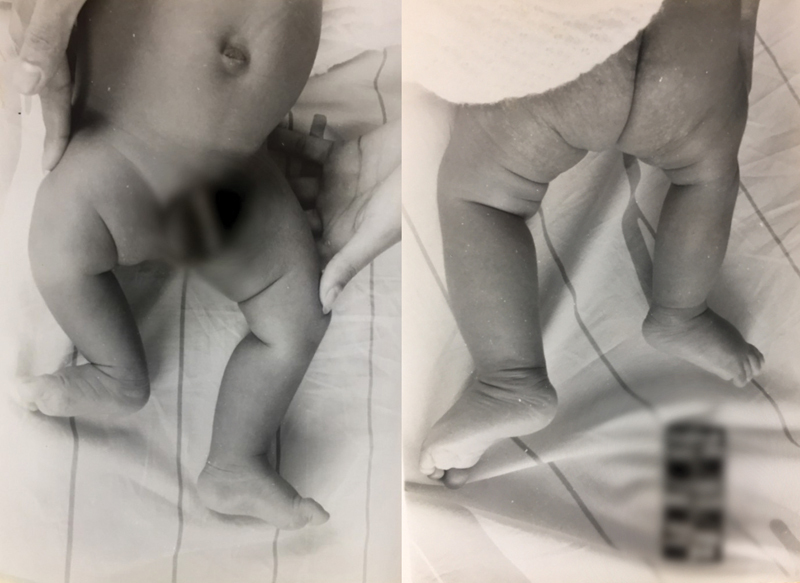
Patient at 35 days of life, presenting discrepancy between the limbs, with the right limb being the shortest.

A patient was evaluated At the Orthopedics Department of Rede Sarah de Hospitais de Reabilitação, in Brasília, the Federal District of Brazil, at 35 days of life due to an abnormal esthetic appearance and shortening of the right lower limb. He was the second child of a healthy mother, the result of an uncomplicated pregnancy, born at term and without identifiable risk factors for congenital anomalies. There were no known relatives with deformity in the limbs or relevant genetic history. The right limb was 3 cm shorter and slightly thinner, and the reduction in length was concentrated in the femur, while the tibias and feet were symmetrical and without deformities ( Fig. 1 ). The hips and knees presented regular and symmetrical range of motion on the contralateral side. There were no findings in the renal, cardiovascular, gastrointestinal and nervous systems; and no upper-limb malformations were observed. The boy had a normal global neuropsychomotor development, and began walking at 14 months.

**Fig. 2:**
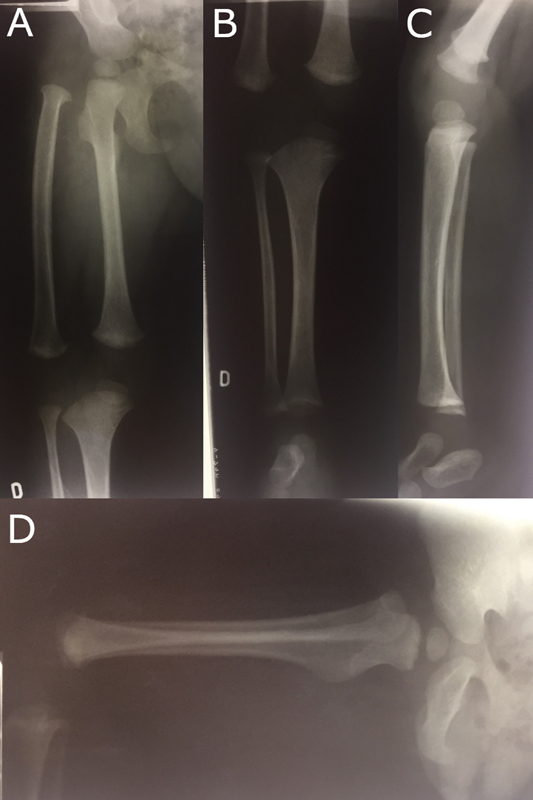
Radiograph at 1 year of age. ( **A** ) Right hip and right femur in anteroposterior (AP) view. ( **B** ) Distal femur and tibia in AP view. ( **C** ) Distal femur and tibia in profile view. ( **D** ) Right hip and femur in profile view.

At 1 year of age, a radiograph showed total duplication of the femur. In the proximal region, the medial femur articulated in the hip without abnormalities, despite a certain valgus of the femoral neck ( Fig. 2 ), and the lateral femur was at the level of the greater trochanter. The proximal epiphysis of the right tibia was hypoplasic and tilted towards the medial femur. The fibula showed no obvious deformities. A radiographic evaluation of the spine showed no deviations or congenital anomalies.

**Fig. 3:**
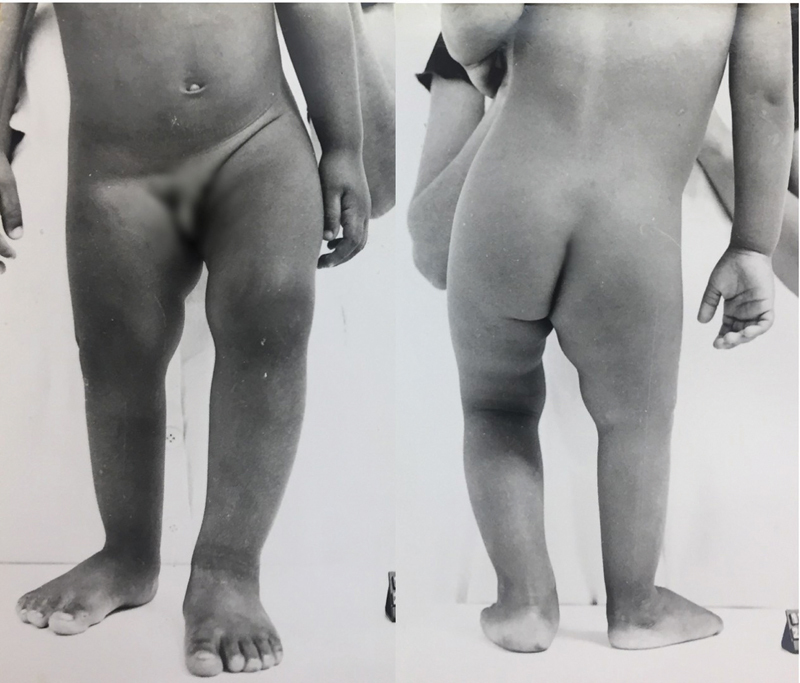
Clinical images of the patient at 3 years of age, showing 6 cm of discrepancy in the length of the legs.

The next consultation took place at the age of 3; at that time, the discrepancy in the lower limbs had increased to 6 cm ( Fig. 3 ). There was a loss in the range of knee flexion on the right side, which was between 0° and 70°. While walking, the patient kept his right foot in equine position (reducible) and his left knee semi-flexed to compensate for the discrepancy.

Radiographs showed the presence of distal epiphysis in the medial femur, and the proximal epiphysis of the tibia kept its development concentrated on the medial side. Using the multiplier method to predict length at 3 years of age, the expected discrepancy would be of 13.4 cm in skeletal maturity. The patient adapted well to a shoe compensation of 5 cm. After this visit, the patient did not return for the next 20 years.

**Fig. 4:**
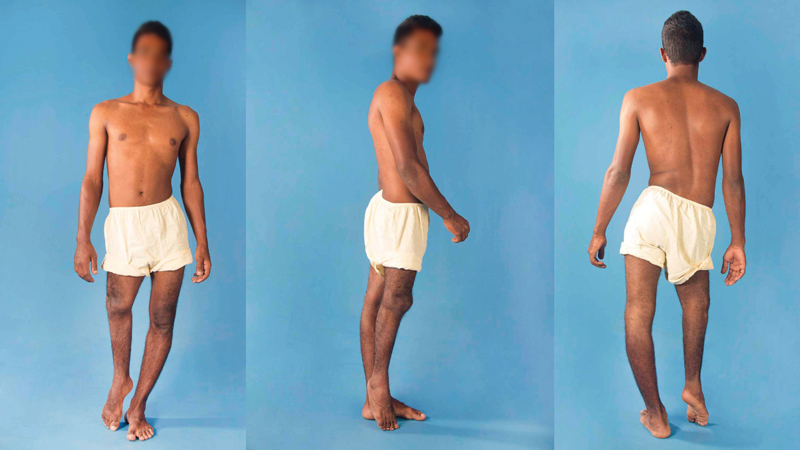
Clinical images of the patient at 23 years of age, showing 17 cm of discrepancy in the length of the legs.

In August 2016, at the age of 23, the patient sought new care. His main concern was the discrepancy in the lower limbs and sporadic low-back pain. He did not use any compensation, and external rotation of the limb was observed at the expense of the hip/femur, and, during gait, he kept his left knee semi-flexed and the right foot in an equine position. In addition, there was evident scoliosis with convexity on the right and pelvic obliquity ( Fig. 4 ). On the right, the flexion-extension motion of the knee ranged from 0° to 95°, and the motion of hip ranged from -20° to 45°, compared to -30° to 90° on the left side.

**Fig. 5:**
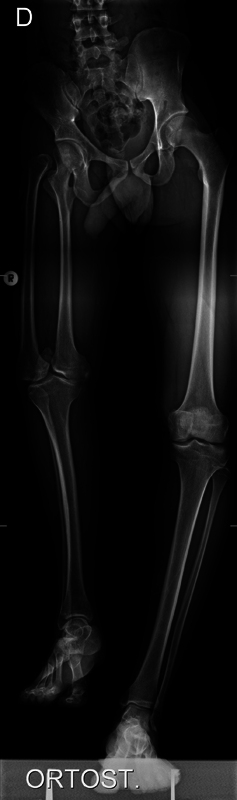
Radiograph in AP view of the lower limbs at 23 years of age. The image shows right-hand valgus femoral neck with normal hip joint, one knee with two separate femoral condyles articulating with the proximal tibia, and a hypolabic patella. The talar dome has a spherical aspect articulating with a concave surface on the tibia, forming a ball-and-socket ankle *.* The right lower limb measures 67 cm (femur: 33 cm; tibia 34 cm), and left limb, 84 cm (femur: 46 cm; tibia 38 cm).

A radiograph showed a femoral neck valgus to the right with a normal hip joint, one knee with two separate femoral condyles that articulated with the proximal tibia, and a hypoplasic patella ( Figure 5 ). The talar dome presented a spherical configuration that articulated with a concave surface in the tibia, forming a ball-and-socket ankle. The radiographic measurements in orthostatism were of 67 cm (femur: 33 cm; tibia: 34 cm) on the right side, and of 84 cm (femur: 46 cm; tibia 38 cm) on the left side, revealing a discrepancy of 17 cm, similar to the clinical measure.

A magnetic resonance imaging (MRI) scan showed femoral condyles with irregular surface, and thin cartilage in the posterolateral part of the lateral condyle and in the anteromedial part of the medial condyle. There are meniscal contours, predominantly in the anterior part of the lateral and posterior compartments of the medial compartment. A low signal structure is identified in intra-articular topography with an oblique path, inserted in the medial face of the lateral femoral condyle and in the posterior tibial plateau.

After receiving a 16-cm orthoprosthesis, there was resolution of the complaint of low-back pain.

## Discussion

The present is the first report in the literature of a case of total duplication of the femur leading to the formation of a peculiar knee. In the images, one can observe the evolution of the condition from the first days after birth until adulthood. Despite the clinical repercussions of the great dysmetria, the patient presented good overall muscle development, and works as an assistant mason, an occupation with a high physical demand. After the use of the orthoprosthesis for three months, the patient reported complete improvement in low-back pain, and was completely asymptomatic at that time. Because of the anatomical structure of the knee, the authors did not consider any surgical approach, as a possible bone elongation could result in significant functional worsening. The treatment with an orthoprosthesis proved to be effective in restoring the patient's quality of life.

There have been few reports of distal femur bifurcation since Erlich's first description in 1889. In general, it occurs associated with other deformities of the ipsilateral limb, such as tibial aplasia, foot hypoplasia, and congenital talipes equinovarus, and it is characteristic of the Gollop-Wolfgang complex. 1 2 3 4 It may also be associated with upper-limb malformations. 5

There are some theories about the etiology of isolated femoral bifurcation, and it is likely to be heterogeneous, as postulated by Bodurtha et al. 6 Embryological neuropathy can lead to a wide variety of abnormalities, depending on the nerves involved and their innervation. The growth of an organ or part of it is related to its nervous supply, due to its co-stimulating function of growth.

Ogden 3 reported a case of femoral bifurcation and ipsilateral tibial hemimelia. He proposed that femoral bifurcation resulted from the division of the mesoderm of the embryonic bud that originates the limb, resulting in an independent development of each of these halves. This division can be produced mechanically by the pressure of amniotic bands, leading to total or partial duplication of the limb.

Osuji et al. 7 reported a patient presenting proximal duplication of the femur who underwent excision of one of the heads and neck of the femur associated with a defeat osteotomy, Salter-type pelvic osteotomy, and adductor tenotomy due to the remaining 70° anteversion of the femoral head. Compared to the present case, complete excision of the femur was not an option, as the lateral part formed the lateral condyle of the femur, and the medial part, the head and neck of the femur.

As this patient was not submitted to surgical intervention, it is possible to observe the natural history of this unusual malformation over 23 years.
