# Supplementary material for: Total Duplication of the Femur: Case Report with 23 Years Follow-up
Source: Rev Bras Ortop (Sao Paulo). 2021 Oct 1;59(5):e801–4. doi: 10.1055/s-0041-1735139 (PMC11624942; doi:10.1055/s-0041-1735139)
Supplement: Related texts

# Research article: Duplicação total do fêmur: Relato de caso com 23 anos de seguimento

**Keywords:** deformidades congênitas dos membros, dismetria, escoliose, fêmur

## Abstract

A duplicação do fêmur é raríssima, e os poucos casos descritos são de duplicações parcial, proximal ou distal. Segundo a nossa revisão de literatura, esse é o primeiro relato de duplicação total. O paciente foi acompanhado, sem tratamento cirúrgico, por 23 anos. Na última visita, queixava-se de dor lombar marcada discrepância dos membros inferiores. A avaliação clínico-radiográfica mostrava um joelho direito com dois côndilos femorais separados que se articulavam com a tíbia proximal e uma patela hipoplásica, e limitação da flexão do joelho em 95°. O paciente apresentava dismetria dos membros inferiores medida em 17 cm, o que resultava em obliquidade pélvica e escoliose dextroconvexa. O paciente recebeu uma ortoprótese de 16 cm que trouxe melhora do padrão de marcha e resolução da queixa de lombalgia.

## Introdução

A deformidade femoral mais comum é o fêmur curto congênito. Alguns autores descreveram raros casos de bifurcação femoral distal, que é característica do complexo de Gollop-Wolfgang, uma síndrome genética rara, geralmente associada a deformidades no membro ipsilateral, tais como aplasia tibial, hipoplasia, ou pé equinovaro. 1 2 3 4 Houve ainda um caso de duplicação femoral proximal relatado em 2009; no entanto, duplicações femorais totais nunca foram descritas na literatura.

## Relato de Caso

Um paciente foi avaliado no setor de ortopedia da Rede Sarah de Hospitais de Reabilitação, em Brasília, aos 35 dias de vida por uma aparência estética anormal e encurtamento do membro inferior direito. Tratava-se do segundo filho de uma mãe saudável, fruto de gravidez sem intercorrências, nascido a termo, e sem fatores de risco identificáveis para anomalias congênitas. Não havia parentes conhecidos com deformidade nos membros ou histórico genético relevante. O membro direito era 3 cm mais curto e levemente mais fino, sendo a redução do comprimento concentrada no fêmur, ao passo que as tíbias e pés eram simétricos e sem deformidades ( Fig. 1 ). Quadris e joelhos apresentavam amplitude de movimento regular e simétrica ao lado oposto. Não houve achados nos sistemas renal, cardiovascular, gastrointestinal e nervoso, e não foram observadas malformações nos membros superiores. O menino apresentou um desenvolvimento neuropsicomotor global normal, e começou a andar aos 14 meses.

**Fig. 1:** Paciente com 35 dias de vida, apresentando discrepância entre os membros, sendo o direito mais curto.

Com 1 ano de idade, uma radiografia mostrou duplicação total do fêmur. Na região proximal, o fêmur medial se articulava no quadril sem anormalidades, apesar de certo valgo do colo femoral ( Fig. 2 ), e o fêmur lateral estava no nível do trocanter maior. A epífise proximal da tíbia direita era hipoplásica e inclinada na direção do fêmur medial. A fíbula não mostrava deformidades evidentes. Uma avaliação radiográfica da coluna vertebral não mostrou desvios ou anomalias congênitas.

**Fig. 2:** Radiografia com 1 ano de idade. ( **A** ) Quadril direito e fêmur direito em incidência anteroposterior (AP). ( **B** ) Fêmur distal e tíbia em incidência AP. ( **C** ) Fêmur distal e tíbia em perfil. ( **D** ) Quadril direito e fêmur em perfil.

O próximo encontro ocorreu aos 3 anos de idade; naquele momento, a disparidade nos membros inferiores havia aumentado para 6 cm ( Fig. 3 ). Observou-se perda na amplitude de flexão do joelho no lado direito, que estava entre 0° e 70°. Enquanto caminhava, o paciente mantinha o pé direito na posição de equino (redutível), e o joelho esquerdo, semiflexionado para compensar a discrepância.

**Fig. 3:** Imagens clínicas do paciente com 3 anos de idade, mostrando uma discrepância de 6 cm no comprimento das pernas.

Radiografias mostraram a presença de epífise distal no fêmur medial, e a epífise proximal da tíbia manteve seu desenvolvimento concentrado no lado medial. Usando o método multiplicador de Paley para a previsão de comprimento aos 3 anos de idade, a discrepância esperada seria de 13,4 cm na maturidade esquelética. O paciente se adaptou bem a uma compensação de sapato de 5 cm. Após essa visita, o paciente não retornou pelos próximos vinte anos.

Em agosto de 2016, aos 23 anos, o paciente buscou novo atendimento. Sua principal preocupação era a discrepância dos membros inferiores e dor lombar esporádica. Ele não usava nenhuma compensação, sendo observada rotação externa do membro às custas do quadril/fêmur, e, durante a marcha, mantinha o joelho esquerdo semi-flexionado e o pé direito em posição equina. Além disso, havia evidente escoliose com convexidade à direita e obliquidade pélvica ( Fig. 4 ). À direita, a amplitude de flexo-extensão do joelho era de 0° a 95°, e a do quadril, de -20° a 45°, comparada a -30° a 90° no lado esquerdo.

**Fig. 4:** Imagens clínicas do paciente com 23 anos, mostrando uma discrepância de 17 cm no comprimento das pernas.

Uma radiografia mostrou um colo femoral valgo à direita, com articulação normal do quadril, um joelho com dois côndilos femorais separados que se articulavam com a tíbia proximal, e uma patela hipoplásica ( Fig. 5 ). O domo do tálus apresentava configuração esférica, articulando-se com uma superfície côncava na tíbia, configurando tornozelo esferoide ( ***tipo ball and socket*** ). As medidas radiográficas em ortostatismo foram 67 cm (fêmur: 33 cm; tíbia: 34 cm) à direita, e 84 cm (fêmur: 46 cm; tíbia: 38 cm) à esquerda, revelando discrepância de 17 cm, semelhante à medida clínica.

**Fig. 5:** Radiografia em AP dos membros inferiores, aos 23 anos de idade. A imagem mostra o colo femoral valgo à direita, com articulação normal do quadril, um joelho com dois côndilos femorais separados se articulando com a tíbia proximal, e uma patela hipoplásica. O domo do tálus tem aspecto esférico, e articula-se com uma superfície côncava na tíbia, configurando tornozelo esferoide ( *tipo ball and socket* ). A medida do membro inferior direito é de 67 cm (fêmur: 33 cm; tíbia 34: cm), e, do esquerdo, de 84 cm (fêmur: 46 cm; tíbia: 38 cm).

Uma ressonância magnética (RM) mostrou côndilos femorais com superfície irregular, e cartilagem fina nas porções posterolateral do côndilo lateral e anteromedial do côndilo medial. Existem contornos de menisco, que predominam nas porções anterior dos compartimentos lateral e posterior do compartimento medial. Uma estrutura de sinal baixo é identificada em topografia intra-articular com trajeto oblíquo, inserido-se na face medial do côndilo femoral lateral e no platô tibial posterior.

Após receber uma ortoprótese de 16 cm, houve resolução da queixa de lombalgia.

## Discussão

Este é o primeiro relato da literatura de uma duplicação total do fêmur levando à formação de um joelho peculiar. Nas imagens, pode-se observar a evolução do quadro, desde os primeiros dias após o nascimento até a idade adulta. Apesar das repercussões clínicas da grande dismetria, o paciente apresentou um bom desenvolvimento muscular global, e trabalha como auxiliar de pedreiro, função com exigência física elevada. Após o uso da ortoprótese por três meses, o paciente referiu melhora completa do quadro de lombalgia, apresentando-se completamente assintomático nesse momento. Por conta da estrutura anatômica do joelho, os autores não consideraram qualquer abordagem cirúrgica, pois um eventual alongamento ósseo poderia resultar em piora funcional significativa. O tratamento com ortoprótese mostrou-se eficaz em restabelecer a qualidade de vida do paciente.

Foram feitos poucos relatos de bifurcação distal do fêmur desde a primeira descrição de Erlich em 1889. De maneira geral, ela ocorre associada a outras deformidades no membro ipsilateral, como aplasia tibial, hipoplasia do pé, e pé equinovaro, e é característica do complexo de Gollop-Wolfgang. 1 2 3 4 Podem também estar associada a malformações dos membros superiores. 5

Há algumas teorias sobre a etiologia da bifurcação femoral isolada, e é provável que seja heterogênea, como postulado por Bodurtha et al. 6 Neuropatia embriológica pode levar a uma ampla variedade de anormalidades, dependendo dos nervos envolvidos e de sua respectiva inervação. O crescimento de um órgão ou parte dele está relacionado ao seu suprimento nervoso, devido à sua função de coestimulador do crescimento.

Ogden 3 relatou um caso de bifurcação femoral e hemimelia tibial ipsilateral. Ele propôs que a bifurcação femoral resultava da divisão do mesoderma do botão embrionário que origina o membro, resultando em um desenvolvimento independente de cada uma dessas metades. Essa divisão pode ser produzida mecanicamente a partir da pressão de bandas amnióticas, levando à duplicação total ou parcial do membro.

Osuji et al. 7 relataram o caso de um paciente que apresentava duplicação proximal do fêmur, e que foi submetido a excisão de uma das cabeças e colo do fêmur associada a uma osteotomia de derrotação, osteotomia pélvica tipo Salter, e tenotomia de adutores devido a anteversão remanescente da cabeça femoral de 70°. Comparado ao caso atual, a excisão completa do fêmur não era uma opção, pois a porção lateral formava o côndilo lateral do fêmur, e a porção medial, a cabeça e o colo do fêmur.

Como esse paciente não foi submetido a intervenção cirúrgica, é possível observar a história natural dessa malformação incomum ao longo de 23 anos.
